# The Impact of Different Levels of Physical Activity on Health among Middle-Aged and Elderly Chinese Adults

**Published:** 2019-11

**Authors:** Jing GUAN, Guojun WANG, Chunli GENG

**Affiliations:** 1. School of Economics, Beijing Technology and Business University, Beijing, China; 2. School of Insurance and Economics, University of International Business and Economics, Beijing, China; 3. Sales Management Department, PICC Property and Casualty Company Limited, Beijing, China

**Keywords:** Physical activity, Self-rated health status, Propensity score matching

## Abstract

**Background:**

There is a lack of specific study of the impact of physical activities on middle-aged and elderly adults in developing countries. We aimed to investigate the causal impact of different levels of physical activity on self-rated health status for Chinese adults with an average age of 61 yr from years 2011 to 2015.

**Methods:**

China Health and Retirement Longitudinal Study (CHARLS) conducted by the Peking University of China was utilized which was a longitudinal database and thus allowed to control for the effect of unobserved individual heterogeneities. In this study, this control was carried out using fixed effect and propensity score matching methods. In addition, this study further took into consideration of the discrete feature of our response variable, self-rated health status, by applying an ordered logit model.

**Results:**

Participating in moderate-to-vigorous physical activity significantly increased individuals’ self-rated health status (*P*<.05) whereas lower intensity physical activity did not increase individuals’ self-rated health status.

**Conclusion:**

Moderate-to-vigorous physical activity is an important instrument to improve the health status of middle-aged and elderly population in China. Government should encourage people to involve in more moderate-to-vigorous physical activity as it is an efficient way to improve individuals’ health status.

## Introduction

Recently, the importance of physical exercise has been emphasized all around the world and many nations have prioritize their efforts in encouraging their people to get involved into physical activities (1–3). For example, the importance of promoting physical activity is acknowledged by the State Council of the People’s Republic of China in the National Fitness Program (2016–2020), aiming to promote physical activity participation nationwide as a way to improve national health status (4). This has also led to increasing attraction to investigate the impact of physical activity on health status among academicians and policy makers.

Physical activity improves individuals’ health though mechanisms of strengthening the immune system and prevention of diseases like depression, diabetes and cancer (1, 5–8), however, these studies were based on experimental intervention research which is generally associated with limitations, namely difficulty in randomizing the treated and control groups, lower number of observations and related ethical or moral issues (9). Therefore, analyzing observational studies is considered to be a good method to draw general conclusions, especially in real world with complex information (10).

Physical activity improves health status for people in Canada (11), West Germany (12), UK (13) and Peru (14). The impact of physical activity depends highly on the intensity of it. For example, higher intensity physical activity is crucial to the health of young people (15, 16). However, previous observational studies had focused on either the adults in developed countries (11–13) or the children in developing countries (14). There is a lack of investigation in the impact of physical activity on health among adults in developing countries.

To address this gap in knowledge, this study examined the impact of different levels of physical activity on health status among middle-aged and elderly adults in China. It is important that China and other developing countries have a better understanding of this relationship as it could help the government to have a clear guidance of proceeding fitness programs among the middle-aged and elderly populations. This could be a potential way to improve their health status and help them to have a better quality of life.

## Methods

### Study design

This study used data from the China Health and Retirement Longitudinal Study (CHARLS) conducted by the Peking University of China. CHARLS was a nationally representative survey which covered 28 provinces in mainland China(There are 31 provinces in mainland China. Xizhang, Hainan and Ningxia provinces were not included into the survey sample.). It targeted individuals more than or equal to 45 years old. In the year 2011, it conducted the baseline survey, and since then a full follow up survey was conducted every other year. The survey conducted in 2015 was the most recent data available.

### Participants

CHARLS provided information at the individual, family and community levels and reported demographic characteristics, family information, health status and living habits information. Our analysis period comprised individuals in the baseline year 2011 and subsequent observations of the same individuals for the years 2013 and 2015. We had 5447 observations in total after dropping ones with missing values.

### Ethics approval

The CHARLS data are publicly available and open to researchers all over the world. The current study is a secondary analysis of the deidentified CHARLS data. Therefore, the researchers have no access to personal identifiers or personal health information that can be linked to individuals while analyzing the data.

### Variables Health status

Our response variable was self-rated health status (SRHS). SRHS measured the individuals’ general health at present, and it was an ordinal indicator taking discrete values from 1 to 5, which corresponded to an individual’s health being self-evaluated as very good, good, fair, poor or very poor, respectively. It is a common measurement for health status and has been used in various related previous studies (17–19).

### Physical activities

Our treatment variables were related to the participation of physical activities (PA). They were binary answers to the questions of whether an individual took vigorous physical activities or moderate physical activities, or walk for at least 10 minutes continuously every week, which were abbreviated to VPA, MPA and WALK. CHARLS defined VPA as activities which made people breathe much harder than normal and might include heavy lifting, digging, plowing, aerobics, fast bicycling, and cycling with a heavy load; MPA as activities which made individuals breathe somewhat harder than normal and might include carrying light loads, bicycling at a regular pace, or mopping the floor; WALK as walking that individuals might do solely for recreation, sport, exercise, or leisure. Similar treatment variables have been used before to distinguish the different intensities of physical activity (20).

### Control variables

Control variables in the present study were age, age square (age^2^), education level, marital status, registration type (hukou), deposit, gender, urban and community activity. These variables could be deemed important determinants of health. Education level was a categorical variable which took values from 1 to 3 depending on whether an individual did not have formal education, did not have bachelor’ degree or had bachelor’ degree, respectively. Marital status was a binary variable which took 1 or 0 depending on whether an individual was married or not. Hukou took values 1 to 4 depending on whether an individual had an agricultural hukou, non-agricultural hukou, unified residence hukou or did not have hukou, respectively. Hukou highly depended on the place people were born and was associated with the living conditions in people’s childhood. In addition, social welfares including types of social insurances were also based on hukou, which could influence individuals’ health conditions (21). Deposit was a continuous variable measuring the amount of Chinese yuan (CNY) an individual had in the bank. Rich people were typically more able to get necessary medical treatments. Gender was a binary variable that took value 1 or 0 depending on whether an individual was male or female. Urban was a binary variable which took 1 or 0 depending on whether an individual lived in an urban area or a rural area. This variable was important because it was observed that the urban areas in general had better medical services and living condition (22). Community activity took value 1 or 0 depending on whether an individual participated in a community-related organization or not. It was included because the interaction with people could influence people’s health (23).

### Data analysis

Our purpose was to estimate the causal impact of different levels of PA on individuals’ health which was measured by SRHS. The statistical software Stata, version 13.0 (Stat Corp. LP, College Station, Texas, USA), was used to analyze the data. It should be noted that individuals’ PA decisions and their SRHS could be affected by unobservable individual characteristics. Therefore, in order to control for them, we considered two methodologies: fixed effect (FE) and propensity score matching (PSM).

Fixed effect (FE) is based on the following regression model No. 1:

$$Y_{it}=\alpha_{1}PA_{it}+\alpha_{2}X_{\mathrm{it}}+\gamma_{i}+\varepsilon_{it},$$

where *Y*_*it*_ is the SRHS for individual *i* in year *t*; *PA*_*it*_ and *X*_*it*_ correspond to our treatment variables and control variables respectively for year *t* and individual *i*. To be more clear, we estimated different equations for different definitions of *PA*_*it*_ regarding *VPA*_*it*_ , *MPA*_*it*_ and *WALK*_*it*_, in order to capture the impact of different levels of PA on SRHS. *γ*_*i*_ is individual fixed effect that controls for unobservable time-invariant characteristics of individuals; *ε*_*it*_ is the error component and *α*_*j*_, where *j=* 1 or 2 are parameters to be estimated. Our focus estimation was *α*_ 1_ which represents the estimate of the causal impact of different levels of PA on individuals’ SRHS.

If there were changes in SRHS due to factors other than PA which were different between treated and control groups, then the FE estimation could be biased. Rosenbaum and Rubin (24) proposed PSM method to reduce this bias in the estimation of treatment effects with observational data sets. Following their method, we applied an observational study to approximate the randomized trial in order to obtain objective causal inference. We obtained similar individuals in the treated and control groups by using the kernel matching method. This approach constructed the counterfactual individual in the control group by giving closer individuals higher weights (25). An individual belonged to the treated group if PA took value 1 while he or she belonged to the control group if PA took value 0. We restricted our sample to common support by deleting all observations with propensity scores (probabilities) larger than the smallest maximum and smaller than the largest minimum of the treated and control groups defined by PA.

It was also necessary to adjust FE and PSM methods from linear cases to non-linear cases as our response variable was coded on a discrete and ordinal scale. Therefore, an ordered logit model was considered to estimate the marginal effects of different levels of PA on SRHS.

## Results

### General characteristics

The characteristics of the 5447 study participants are summarized in Table 1. Our samples were mainly middle-aged and elderly people with an average age of 61. 49% of participants were male. 28% of participants were living in urban areas.

**Table 1:** Statistical description of variables

***Variable***	***Mean***	***Standard Deviation***	***Minimum***	***Maximum***
SRHS	2.91	0.93	1	5
VPA	0.35	0.48	0	1
MPA	0.55	0.50	0	1
WPA	0.80	0.40	0	1
Age	61.21	9.20	45	105
Age^2^	3830.81	1174.38	2025	11025
Education	1.76	0.48	1	3
Marriage	0.82	0.38	0	1
Hukou	1.20	0.41	1	4
Deposit (CNY)	11262.09	55769.23	0	2000000
Gender	0.49	0.50	0	1
Urban	0.28	0.45	0	1
Community	0.02	0.15	0	1

### Linear analysis of PA for SRHS

Table 2 shows the estimated impact of different levels of PA on individuals’ SRHS for the two above discussed approaches: FE and PSM. For the sake of brevity, only the estimations of the focus parameters are reported. Under the PSM method, the average treatment effect estimation (ATE) is presented. Columns 2, 4 and 6 show the results from the FE estimation for VPA, MPA and WALK, respectively and columns 3, 5 and 7 present PSM estimations for VPA, MPA and WALK, respectively.

**Table 2:** Impact of different levels of PA on individuals’ SRHS

	***VPA***		***MPA***		***WALK***	
	***FE***	***PSM***	***FE***	***PSM***	***FE***	***PSM***
PA	−0.066 (−0.99)	−0.078 ^***^ (−2.80)	−0.158 ^***^ (−2.82)	0.099 ^***^ (−3.77)	−0.043 (−0.55)	−0.048 (−1.46)
R-square	0.014	0.002	0.025	0.003	0.013	0.001
#Obs.	5447	5446	5447	5435	5447	5410

Notes: T statistics in parentheses.

* *P*<0.1

** *P* <0.05

*** *P* <0.01. The number of observations was less than 5447 under PSM approach. This is because we restricted our sample to common support

Both estimation methods consistently showed that MPA significantly increased individuals’ SRHS. To be more specific, MPA significantly increased individual’s SRHS between 0.099 to 0.158 units using PSM and FE methods, respectively. We also found some evidence that VPA could increase individuals’ SRHS by 0.078 unites, however, the estimation was only significant under PSM method and not for FE method. We found that WALK did not provide a significant improvement on the SRHS. This could due to the reason that walking requires very few physical efforts and thus do not have a significant effect on individuals’ SRHS. It was important to test if our control variables achieve good balance after matching under PSM method (26). As it was discussed in the Methods section, the changes in SRHS should not due to factors other than PA, therefore, the control variables should not be significantly different between the treated and control groups after matching. One way to test good balance is to check the standardized mean differences of covariates between the two groups after balancing.

Under three specifications regarding VPA, MPA and WALK, the standardized mean differences were less than 10 in all cases which suggested that our estimations passed the balancing test(PSM balance estimations are available from the authors upon request) (26).

### Non-linear analysis of PA for SRHS

Given the fact that our response variable was coded on a discrete and ordinal scale, a nonlinear model was especially desired under this context (27). For the robustness of the results, we carried on our analysis by using an ordered logit model with PSM method. The reason we did not continue with this non-linear model for FE method was because there is no simple transformation available that would purge the ordered response models from the individual fixed effect (27). And we could include the individual fixed effect unless we collapsed the 5 categorical responses into two classes and applied a binary logit model (27–29).

Table 3 presents the marginal effects at means of different levels of PA on SRHS using an ordered logit model with PSM method.

**Table 3:** Marginal effects at means of the impact of different levels of PA on individuals’ SRHS using an ordered logit model with PSM method

***Variable***	***Very good***	***Good***	***Fair***	***Poor***	***Very poor***
VPA	0.017 ^***^ (3.17)	0.016 ^***^ (3.16)	−0.004 ^**^ (−2.40)	−0.022 ^***^ (−3.19)	−0.007 ^***^ (−3.16)
MPA	0.019 ^***^ (3.98)	0.019 ^***^ (3.90)	−0.004 ^***^ (−3.10)	−0.026 ^***^ (−3.87)	−0.009 ^***^ (−3.73)
WALK	0.010 ^*^ (1.67)	0.009 (1.63)	−0.001 ^**^ (−1.98)	−0.013 (−1.60)	−0.005 (−1.56)

Notes: Z statistics in parentheses.

* *P*<0.1

** *P* <0.05

*** *P* <0.01

The results showed that VPA and MPA both significantly increased the probability of individuals reporting their SRHS as very good (1.7 pp and 1.9pp for VPA and MPA, respectively) and good (1.6pp and 1.9pp for VPA and MPA, respectively), where pp indicates percentage points. In addition, VPA and MPA significantly decreased the probability of individuals reporting their SRHS as fair (0.4pp for both VPA and MPA), poor (2.2pp and 2.6pp for VPA and MPA, respectively) and very poor (0.7pp and 0.9pp for VPA and MPA, respectively). It could be seen that MPA had a larger impact on SRHS comparing to VPA. However, the impact of WALK was not significant in the second (good), fourth (poor) and fifth (very) levels, and only marginally significant in the first (very good) level. This was consistent with our linear estimations that moderate-to-vigorous PA had a significant positive impact on individuals’ SRHS.

## Discussion

This paper presented novel findings on the impact of different levels of PA on the SRHS of Chinese adults with an average age of 61 years old. We found a strong evidence that moderate-to-vigorous PA significantly improved the SRHS among Chinese middle-aged and elderly adults between 0.078 to 0.158 units using various methods. After taking into consideration of the discrete feature of our response variable, moderate-to-vigorous PA significantly increased the probability of individuals reporting their SRHS as very good and good by 1.7–1.9pp and 1.6–1.9pp, respectively. In addition, moderate-to-vigorous PA significantly decreased the probability of individuals reporting their SRHS as fair, poor and very poor by 0.4pp, 2.2–2.6pp and 0.7–0.9pp, respectively. However, we did not find evidence that WALK significantly improved individuals’ SRHS. Present results are consistent with previous literature where PA improved individuals’ health status (11–14), especially high intensity of PA was associated with good health (15) and low all-cause mortality (16). For example, Humphreys et al (11) used sample from residents over 12 years old in Canada at the year 2005 and found a significant positive impact of PA, especially moderate PA, on individuals’ health outcomes. Lechner (12) investigated the impact of leisure sports participation on health based on individual samples from Germany during the year 1984 to 2006 and found a positive effect of sports participation on health. Rasciute and Downward (13) investigated the impact of physical activities such as sports participation and active forms of transport on self-rated health using a three-year survey in England which was commenced in 2005 and a broadly positive effect upon health was found. Pawlowski et al (14) studied the children in Peru at the years 2002, 2006 and 2009 and also found similar positive impact of PA on health. Driediger et al (15) mentioned the importance of carrying out PA among Canada and Australia children in their opinion research. Samitz et al (16) conducted a systematic review on the similar topic and found risk reduction per unit of time increase was largest for vigorous exercise. Previous research for developed countries (11–13, 15) and children (14) cannot be applied for adults in developing countries, because other than the variances of methods and survey periods, their economic level, health stock, sports involvement and physical education were also different (30, 31).

As stated before, there was still a scarcity of research for relating physical activity to health among middle-aged and elderly population in developing countries (32, 33). This study was the first longitudinal study which investigated the impact of different levels of PA on SRHS among this population. China, a developing and the most populated country in the world, was a good start to study developing country issues. Our results indicated that participating in PA is an important instrument to improve individuals’ health. In addition, the intensities of PA play important roles. Individuals could benefit more from moderate-to-vigorous PA. One potential explanation for the different impacts of various levels of PA could be based on the previous studies where it was shown that participating in different intensities of PA were related to individuals’ different levels of aerobic capacities (34) and amounts of insulin (35). The negligible impact of the low intensity PA, such as walking, on SRHS could due to the less requirements of physical effort. As also suggested by the UK National Health Service (NHS), middle-aged and elderly adults should do two types of physical activities: aerobic exercise and strength exercise (2, 3).

There are three main contributions of the present study. First, to the best of our knowledge, this was the first longitudinal study to analyze the impact of physical activities on health among middle-aged and elderly adults in a developing country. Second, relationships of different levels of physical activity (or intensities of physical efforts) to the health benefits were made. Third, a comprehensive longitudinal database CHARLS was used in the present study which allowed us to control for the presence of time-invariant effects by observing the same person before and after being involved in physical activities. Moreover, to avoid the effect of potential endogeneity between physical activity and SRHS, propensity score method was employed in the present study (24). The limitation of this study is the health status variable used in this study was self-reported, this might result to the reporting bias (36). We were not able to detect the impact of PA on objective health status indicators, for example, individuals’ health information from the hospitals, due to data limitation. This kind of study is specially warranted in the future.

There are public health implications of this study. For example, with the increasing proportion of elder population in China (33), as well as in the world, a cheap and effective way to improve the health condition in the aging society is necessary. Our study not only offered the evidence that PA improves health status among middle-aged and elderly adults in China, but also presented the clear way of proceeding the health-related policy by emphasizing the benefits and importance of moderate-to-vigorous PA. Our recommendation is that the government should encourage people to involve in more moderate-to-vigorous physical activities, for example aerobics, as they are efficient ways to improve individuals’ health status.

## Conclusion

Moderate-to-vigorous physical activities significantly improve the individuals’ self-rated health status in Chinese middle-aged and elderly adults. However, walking, a lower intensity of physical activity, has a very less impact on the improvement of self-rated health status.

## Ethical considerations

Ethical issues (Including plagiarism, informed consent, misconduct, data fabrication and/or falsification, double publication and/or submission, redundancy, etc.) have been completely observed by the authors.

## References

[1] Warburton DE Nicol CW Bredin SS (2006). Health benefits of physical activity: the evidence. CMAJ, 174( 6): 801–9. 16534088 10.1503/cmaj.051351 PMC1402378

[2] NHS (2018). Physical activity guidelines for older adults https://www.nhs.uk/live-well/exercise/physical-activity-guidelines-older-adults/

[3] NHS (2018). Physical activity guidelines for adults https://www.nhs.uk/live-well/exercise/

[4] The State Council of the People's Republic of China (2016). National fitness program (2016–2020) . http://www.gov.cn/zhengce/content/2016-06/23/content_5084564.htm (in Chinese)

[5] Sherwood NE Jeffery RW (2000). The behavioral determinants of exercise: implications for physical activity interventions. Annu Rev Nutr, 20( 1): 21–44. 10940325 10.1146/annurev.nutr.20.1.21

[6] Brown WJ Burton NW Rowan PJ (2007). Updating the evidence on physical activity and health in women. Am J Prev Med, 33(5): 404–11. e25. 17950406 10.1016/j.amepre.2007.07.029

[7] Tsang HW Chan EP Cheung WM (2008). Effects of mindful and non-mindful exercises on people with depression: a systematic review. Br J Clin Psychol, 47( 3): 303–22. 18237457 10.1348/014466508X279260

[8] Janssen I LeBlanc AG (2010). Systematic review of the health benefits of physical activity and fitness in school-aged children and youth. Int J Behav Nutr Phys Act, 7( 1): 40. 20459784 10.1186/1479-5868-7-40 PMC2885312

[9] Faraoni D Schaefer ST (2016). Randomized controlled trials vs. Observational studies: why not just live together? BMC Anesthesiol, 16 ( 1 ): 102 . 27769172 10.1186/s12871-016-0265-3 PMC5073487

[10] Schillaci G Battista F Pucci G (2013). Are observational studies more informative than randomized controlled trials in hypertension? Hypertension, 62 ( 3 ): 470 – 6 . 23876470 10.1161/HYPERTENSIONAHA.113.01501

[11] Humphreys BR McLeod L Ruseski JE (2014). Physical activity and health outcomes: evidence from canada. Health Econ, 23( 1): 33–54. 23364850 10.1002/hec.2900

[12] Lechner M (2009). Long-run labour market and health effects of individual sports activities. J Health Econ, 28( 4): 839–54. 19570587 10.1016/j.jhealeco.2009.05.003

[13] Rasciute S Downward P (2010). Health or happiness? What is the impact of physical activity on the individual? Kyklos, 63 ( 2 ): 256 – 70 .

[14] Pawlowski T Schüttoff U Downward P Lechner M (2018). Can sport really help to meet the millennium development goals? Evidence from children in peru. J Sports Econom, ( No.4 ): 498 – 521 .

[15] Driediger M Vanderloo LM Truelove S Bruijns BA Tucker P (2018). Encouraging kids to hop, skip, and jump: Emphasizing the need for higher-intensity physical activity in childcare. J Sport Health Sci, 7( 3): 333–6. 30356615 10.1016/j.jshs.2018.03.003 PMC6189234

[16] Samitz G Egger M Zwahlen M (2011). Domains of physical activity and all-cause mortality: Systematic review and dose-response meta-analysis of cohort studies. Int J Epidemiol, 40( 5): 1382–400. 22039197 10.1093/ije/dyr112

[17] Gu LB Feng HH Jin J (2017). Effects of medical insurance on the health status and life satisfaction of the elderly. Iran J Public Health, 46( 9): 1193–203. 29026784 PMC5632320

[18] Chiao C Ksobiech K Wei CY (2014). National health insurance and life satisfaction in late life: Longitudinal findings from a natural experiment in taiwan. J Public Health (Oxf), 36( 2): 308–16. 23735959 10.1093/pubmed/fdt055

[19] Tamiya N Noguchi H Nishi A (2011). Japan: Universal health care at 50 years 4 population ageing and wellbeing: Lessons from japan's long-term care insurance policy. Lancet, 378( 9797): 1183–92. 21885099 10.1016/S0140-6736(11)61176-8

[20] O'Donovan G Blazevich AJ Boreham C Cooper AR (2010). The abc of physical activity for health: A consensus statement from the british association of sport and exercise sciences. J Sports Sci, 28( 6): 573–91. 20401789 10.1080/02640411003671212

[21] Li X Zhang W (2013). The impacts of health insurance on health care utilization among the older people in China. Soc Sci Med, 85 : 59 – 65 . 23540367 10.1016/j.socscimed.2013.02.037

[22] Deng YZ Paul DR (2018). The relationships between depressive symptoms, functional health status, physical activity, and the availability of recreational facilities: a rural-urban comparison in middle-aged and older chinese adults. Int J Behav Med, 25( 3): 322–30. 29498014 10.1007/s12529-018-9714-3

[23] Johnson BT Acabchuk RL (2018). What are the keys to a longer, happier life? Answers from five decades of health psychology research. Soc Sci Med, 196: 218–26. 29153315 10.1016/j.socscimed.2017.11.001 PMC6894515

[24] Rosenbaum PR Rubin DB (1983). The central role of the propensity score in observational studies for causal effects. Biometrika, 70( 1): 41–55.

[25] Mensah J Oppong JR Schmidt CM (2010). Ghana's national health insurance scheme in the context of the health mdgs: an empirical evaluation using propensity score matching. Health Econ, 19 ( S1 ): 95 – 106 . 20730999 10.1002/hec.1633

[26] Rosenbaum PR Rubin DB (1985). Constructing a control group using multivariate matched sampling methods that incorporate the propensity score. Am Stat, 39( 1): 33–8.

[27] Baetschmann G Staub KE Winkelmann R (2015). Consistent estimation of the fixed effects ordered logit model. J R Stat Soc Ser A Stat Soc, 178( 3): 685–703.

[28] Winkelmann L Winkelmann R (1998). Why are the unemployed so unhappy?Evidence from panel data. Economica, 65( 257): 1–15.

[29] Chamberlain G (1979). Analysis of Covariance with Qualitative Data . National Bureau of Economic Research Cambridge , Mass, USA .

[30] NHS Digital (2017). Statistics on obesity, physical activity and diet. Available from: https://assets.publishing.service.gov.uk/government/uploads/system/uploads/attachment_data/file/613532/obes-phys-acti-diet-eng-2017-rep.pdf

[31] Andreff W (2001). The correlation between economic underdevelopment and sport. Eur Sport Manag Q, 1( 4): 251–79.

[32] Li F Liu Y Harmer PA (2016). Physical activity, aging, and health in China: Addressing public health needs in the presence of continued economic growth and urbanization. J Sport Health Sci, 5( 3): 253–4. 30356487 10.1016/j.jshs.2016.06.009 PMC6188604

[33] Zhu W Chi A Sun Y (2016). Physical activity among older Chinese adults living in urban and rural areas: a review. J Sport Health Sci, 5( 3): 281–6. 30356525 10.1016/j.jshs.2016.07.004 PMC6188614

[34] Rognmo Ø Hetland E Helgerud J Hoff J Slørdahl SA (2004). High intensity aerobic interval exercise is superior to moderate intensity exercise for increasing aerobic capacity in patients with coronary artery disease. Eur J Cardiovasc Prev Rehabil, 11( 3): 216–22. 15179103 10.1097/01.hjr.0000131677.96762.0c

[35] Schwarz AJ Brasel JA Hintz RL Mohan S Cooper DM (1996). Acute effect of brief low- and high-intensity exercise on circulating insulin-like growth factor (IGF) I, II, and IGF-binding protein-3 and its proteolysis in young healthy men. J Clin Endocrinol Metab, 81( 10): 3492–7. 8855791 10.1210/jcem.81.10.8855791

[36] Tong Y Jun M Yan JIANG JL Yunlong Gen YW Wenjie S (2018). Assessing pain among Chinese elderly-chinese health and retirement longitudinal study. Iran J Public Health, 47( 4): 553–60. 29900140 PMC5996333

